# Purpura Fulminans as a Rare Manifestation of Invasive Haemophilus influenzae Disease: A Case Report

**DOI:** 10.7759/cureus.55016

**Published:** 2024-02-27

**Authors:** Diana Oliveira Miranda, Maria Bourbon Ruão, José Magalhães, Nuno Miguel Pereira, Celina Gonçalves

**Affiliations:** 1 Medicine, Centro Hospitalar Universitário de Santo António, Porto, PRT; 2 Critical Care Medicine, Centro Hospitalar Universitário de Santo António, Porto, PRT; 3 Medicine, Centro Hospitalar Universitário do Porto, Porto, PRT

**Keywords:** immunocompetent patients, acute epiglottitis in adults, haemophilus influenzae type b, invasive haemophilus influenzae disease, purpura fulminans

## Abstract

*Haemophilus influenzae *(Hi) is a bacterium usually found in the upper respiratory tract of humans. Though it is recognized as a naturally occurring element in the human bacterial reservoir, Hi infections have the potential to be severe and even fatal, particularly when they result in conditions such as meningitis or epiglottitis. Because of this, Hi invasive infections are considered a reportable disease in Portugal.

We report a case of a 58-year-old female, chronically adrenally suppressed on long-term steroids, who developed an invasive Hi type b infection that led to purpura fulminans and multiorgan failure after an acute episode of epiglottitis. According to our review of the literature, only three previous cases of invasive Hi type b disease-causing purpura fulminans have been described.

## Introduction

*Haemophilus influenzae* (Hi) is an aerobic Gram-negative coccobacillus that makes its entrance into the body through the nasopharynx, colonizing that area. There are two main categories of Hi: encapsulated and non-encapsulated types. The encapsulated bacterium is further segmented into six different subtypes "a" through "f," distinguished by capsule type. Among these, the most recognizable and prevalent form is Hi type b (Hib). Before vaccination programs, Hib could be detected in the nasopharynx of 0.5% to 3% of children under five years of age, representing approximately 41 per 100,000 in Europe. Four years after the introduction of Hib conjugate vaccines in Europe, the incidence of Hib invasive disease had decreased by 97% [1,2,3]. Bloodstream infection by this pathogen can lead to severe, life-threatening complications, including purpura fulminans, an infrequent complication linked to bacterial or viral infections, characterized by extensive hemorrhaging into the skin, mucous membranes, and occasionally the adrenal glands [4]. This condition, often accompanied by disseminated intravascular coagulation (DIC) and shock, has been documented as a result of infections such as varicella, rubella, scarlet fever, meningococcaemia, and Haemophilus influenzae infection [5]. A distinctive feature of purpura fulminans is the emergence of ischemic necrosis in the extremities, attributed to the obstruction of small blood vessels by microthrombi and tissue edema. 

The rarity of our case emphasizes the need for increased awareness and attentiveness in the diagnosis and management of severe complications linked to Hi infections. Recognizing these uncommon yet crucial manifestations plays a key role in advancing clinical knowledge and highlights the continued significance of investigating and documenting cases. This contributes to a deeper understanding of the potential complications associated with Hi, reinforcing the importance of ongoing studies and case reporting.

## Case presentation

Admission

We present a 58-year-old female with a medical history of type 2 diabetes, obesity (having undergone gastric bypass surgery two years prior to this episode), and non-specific arthritis, necessitating a prolonged course of 5mg/day prednisolone over a period exceeding 20 years. A few weeks preceding her hospital admission, her grandson was diagnosed with acute tonsillitis. Upon admission to the emergency room (ER), the patient reported symptoms including fever, hoarseness, cough, and dyspnea over the preceding 48 hours. Additionally, she expressed discomfort in the oropharynx, describing a sensation akin to a foreign body, along with difficulty in vocalization.

Evolution in the ER

A few hours after her admission to the ER, the patient developed severe hypotension unresponsive to intravenous resuscitation, hyperlactatemia, oliguria (with subsequent metabolic acidosis), along with lethargy and stridor, manifesting as acute respiratory dysfunction and hypercapnia, thus evolving to septic shock. Fibroscopy showed acute epiglottitis with edema of the supraglottic structures leading to orotracheal intubation. Blood cultures and respiratory secretions (RS) cultures isolated multisensitive Hi II. Antibiotic therapy was started with ceftriaxone 2 g/daily, which the patient completed for 10 days, with sterilization proven in two sets of blood cultures and RS. 

Evolution after ICU admission

Due to the severity of the condition, the patient was transferred to the ICU. The results of the analytical study conducted upon admission are displayed in Table 1.

**Table 1:** Results of the patient's blood tests upon admission to the ICU PT - prothrombin time; aPTT - activated partial thromboplastin time; INR - international normalized ratio

Parameters	Values	Reference range
White blood cells	71.21	4.8 – 10.8 (x 10^3/µL)
Neutrophils	64.94	2.00 - 7.50 (x 10^3/µL)
Eosinophils	0.00	0.00 – 0.49 (x 10^3/µL)
Basophils	0.00	0.0 – 0.1 (x 10^3/µL)
Lymphocytes	1.71	1.0 – 4.8 (x 10^3/µL)
Monocytes	1.35	0.12 – 0.80 (x 10^3/µL)
Platelets	30000	150 – 350 (x 10^3/µL)
Hemoglobin	7.8	12 – 15 (g/dL)
C-reactive protein	518.53	<3.0 (mg/dL)
Urea	69	15 – 39 (mg/dL)
Creatinine	4.09	0.70 – 1.30 (mg/dL)
Sodium	135	135 – 146 (mEq/L)
Potassium	4.9	3.5 – 5.1 (mEq/L)
Total bilirubin	0.43	0.3 – 1.2 (mg/dL)
Aspartate aminotransferase	2280	12 – 40 (UI/L)
Alanine aminotransferase	1014	7 – 40 (UI/L)
Gamma-glutamyl transferase	613	0 – 73 (UI/L)
Alkaline phosphatase	691	46 – 116 (UI/L)
D-dímers	>5250	<500 (ng/mL)
PT	23.1	11.4 (seconds)
aPTT	60.4	26.9 (seconds)
Fibrinogen	7.3	2.0 - 4.0 g/L
INR	2.0	1.5

Respiratory Dysfunction

Slow ventilatory weaning led to the need for a tracheostomy performed three weeks after admission, without complications. Prolonged ventilatory weaning was required due to muscle weakness developed during hospitalization, associated with nosocomial respiratory infection with *Enterobacter cloacae*, treated with seven days of trimethoprim/sulfamethoxazole. Successful decannulation occurred after one month, with ongoing follow-up and regular speech therapy. 

Cardiovascular Dysfunction

An echocardiogram showed mild depression/preserved left ventricular ejection fraction (LVEF), without dilation of the right cavities and symmetric systolic flow in the pulmonary artery (PA), with a 14 mm inferior vena cava (IVC). The favorable and rapid evolution of the cardiovascular dysfunction allowed complete weaning off norepinephrine and dobutamine by day 6 of hospitalization.

Renal Dysfunction

Upon admission with oliguria requiring daily sustained low-efficiency dialysis (SLEDD) for two months, followed by gradual recovery of diuresis under significant diuretic stimulation. Maximum creatinine 6.0 mg/dL. Upon discharge from the ICU, the patient had preserved diuresis (>1 mL/kg/h) without diuretic stimulation, but with metabolic acidosis requiring bicarbonate replacement (1000 mg twice daily) to correct the acid-base disturbance, and creatinine at 4.74 mg/dL.

Hepatic Dysfunction

Rapid and favorable evolution of hepatic dysfunction within three weeks of admission.

Coagulopathy

**Figure 1:**
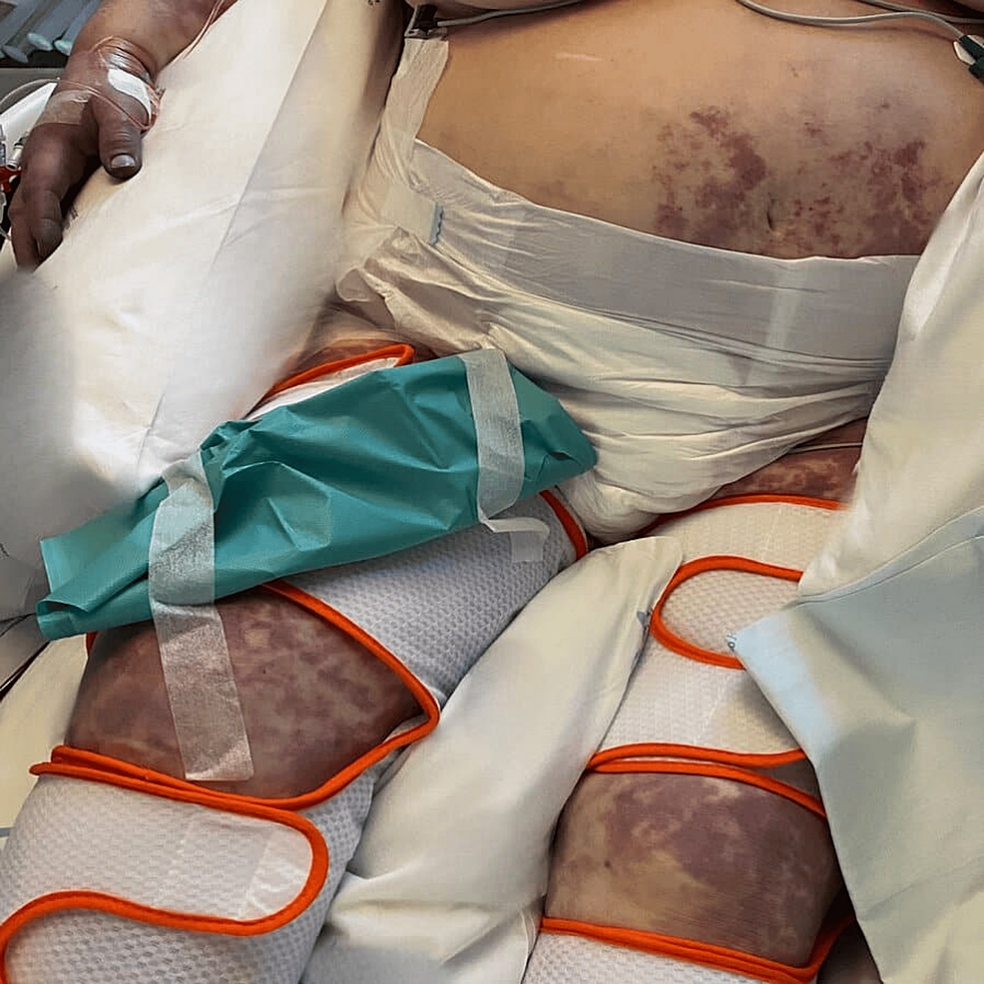
Purpura fulminans on bilateral legs and torso

**Figure 2:**
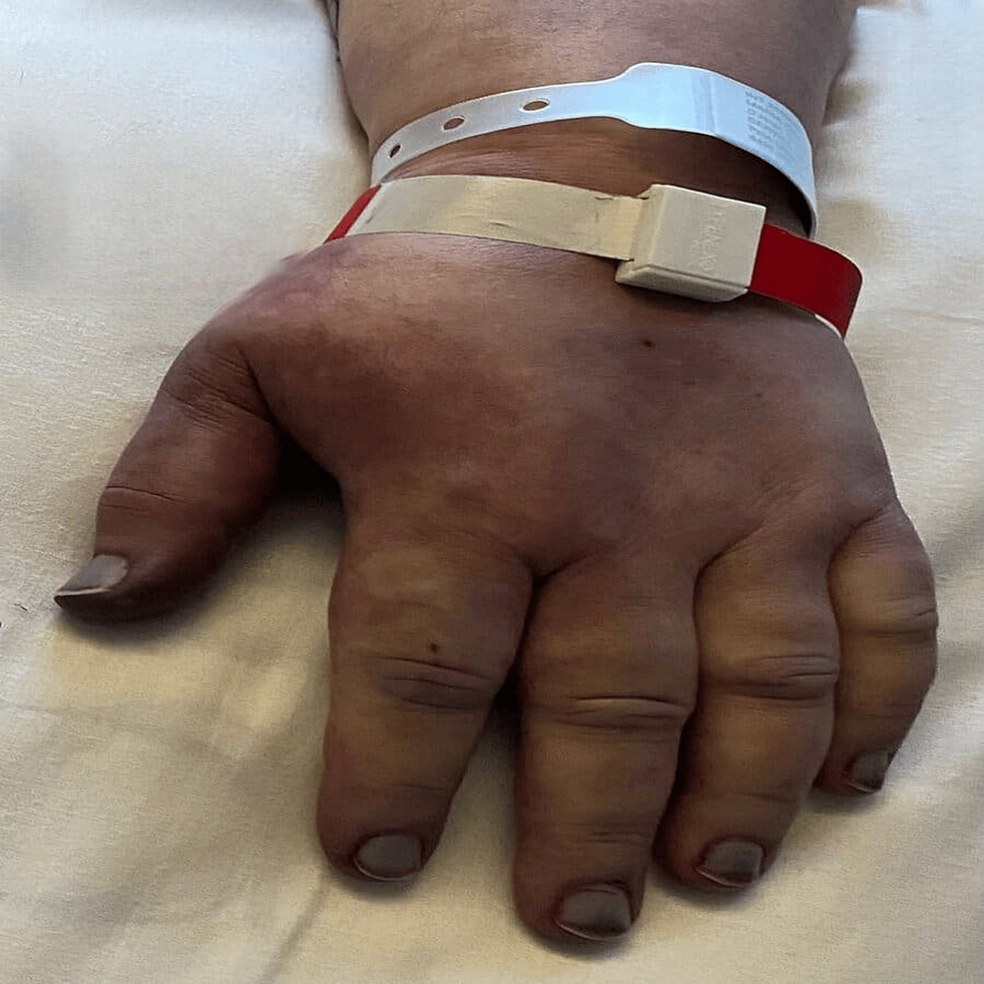
Purpura fulminans on the hand

**Figure 3:**
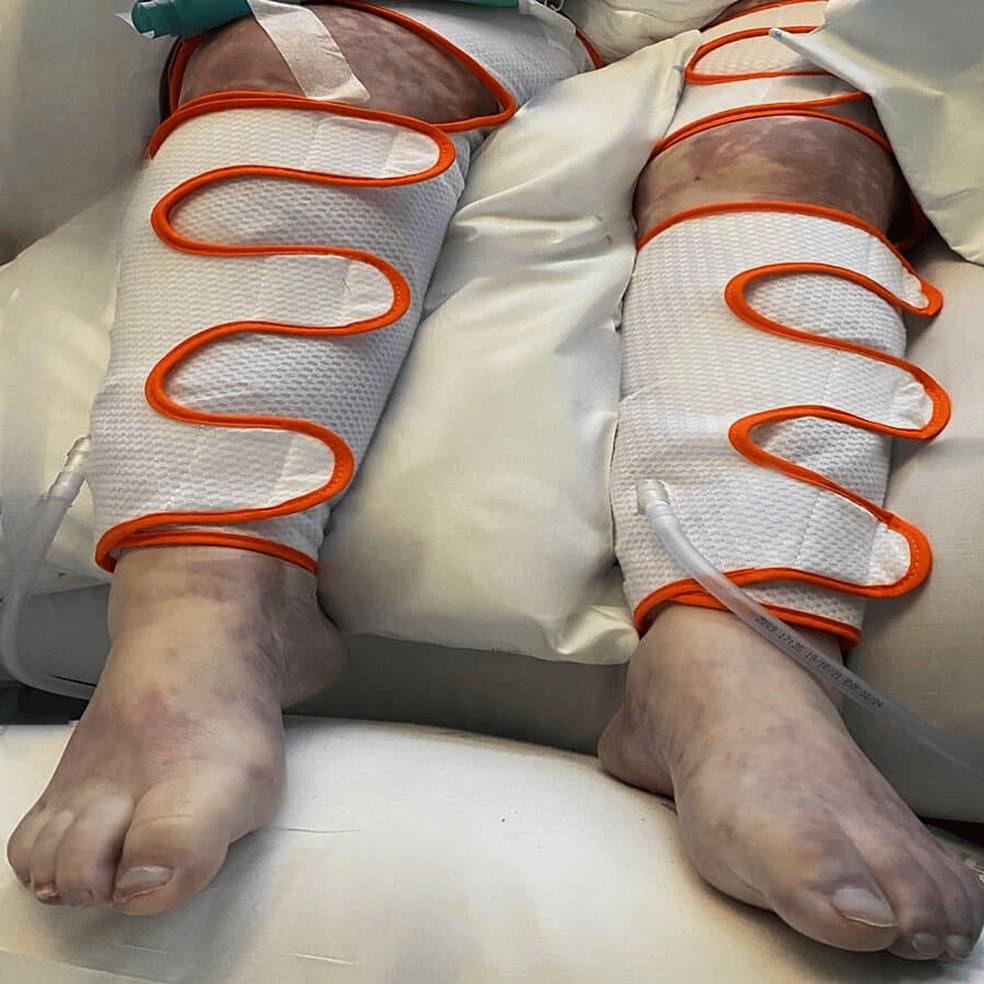
Purpura fulminans on lower limbs

Since being admitted, the patient exhibited criteria indicative of disseminated intravascular coagulation, with the manifestation of purpuric lesions on the distal extremities of all limbs evolving to dry necrosis, leading to the diagnosis of purpura fulminans (Figures 1, 2, 3). Multidisciplinary discussions with Orthopedics, Plastic Surgery, and Vascular Surgery resulted in the decision for phased amputation of the limbs due to tissue non-viability. The patient underwent a transtibial amputation of the left lower limb and transradial and cubital amputation of the right upper limb two weeks after admission, followed by a transtibial amputation of the right lower limb + transradiocubital amputation of the left upper limb two months after admission, both procedures without complications. 

Subsequently, she was discharged from the ICU after three months of hospitalization and transferred to the vascular surgery ward, for optimization of medical therapy and physical rehabilitation.

## Discussion

As one of the few documented cases of purpura fulminans resulting from invasive Hi infection originating from epiglottitis in the adult population, it is essential to emphasize the severity of such infections in a post-vaccination era, especially in adult patients, in which the clinical presentation may deviate from the conventional patterns observed in the pediatric population [6,7]. The epidemiological landscape of invasive Hi disease has undergone notable changes after the widespread vaccination implementation, as Hib previously accounted for over 90% of Hi-invasive diseases, with a remarkable reduction in both infection and colonization [2]. In Portugal, this vaccine has been accessible since 1994, becoming part of the National Vaccination Program (NVP) in 2000 [8]. 

Epiglottitis is characterized by infection and inflammation of the epiglottis, the throat tissue responsible for covering and safeguarding the larynx during swallowing. This condition has the potential to lead to a life-threatening airway obstruction. The above case presentation has many features that are consistent with a common presentation of typical epiglottitis, namely the presence of fever, stridor, respiratory distress, sore throat, painful swallowing, altered voice, and cough [9]. 

It is known that the coagulation cascade is affected during the occurrence of sepsis and septic shock, resulting in the consumption of platelets and clotting factors. This can potentially manifest as disseminated intravascular coagulation (DIC), which presents with a broad spectrum of manifestations. Among these is purpura fulminans, an uncommon and potentially fatal condition, characterized by typical mucocutaneous lesions, resulting from a profound imbalance in the coagulation processes in the microcirculation and leading to thrombotic and hemorrhagic phenomena. In adults, purpura fulminans commonly develops in the presence of coagulopathy induced by septic conditions. There are proposals regarding the mechanism of action of this entity, namely deficiencies of protein C, protein S, and antithrombin. Proteins C and S act as natural anticoagulants produced by the liver, relying on vitamin K for synthesis [3,6,10]. This malfunction of the protein C pathway may be due to reduced liver production in the presence of liver dysfunction and/or acute depletion during coagulation activation [10-12]. It is also still unclear which factors determine which patients will present thrombosis in the presence of an process autoimmune in the context of a coagulopathy or infection [3,11,13]. In a study conducted by Lerolle et al., it was revealed that patients suffering from sepsis and purpura fulminans had decreased levels of protein S and antithrombin compared to those with sepsis alone and those with sepsis and DIC [5].

As previously mentioned, our patient fulfilled the criteria for DIC and liver failure, being in a prothrombotic state that resulted in the development of purpura fulminans. Although some cases of purpura fulminans have been reported in association with Hi infections, the intricate interplay of host factors, microbial virulence, and the immune response leading to it warrants further elucidation.

The primary objective in treating purpura fulminans is to halt its progression, avoid secondary infections, eliminate non-viable tissue, and offer rehabilitation services, considering the potential suitability of hyperbaric oxygen therapy in specific instances, and when available, as recent insights propose this therapy as a potential means to prevent or reverse ischemic damage and the need for amputation [6]. 

This case serves as an illustration of the rare occurrence of purpura fulminans as a manifestation of sepsis due to invasive Hib disease, either independently or in conjunction with pre-existing coagulation abnormalities, whether innate or acquired. 

## Conclusions

Our case report highlights the uncommon yet critical involvement of *Haemophilus influenzae* (Hi) in the pathophysiology of purpura fulminans. Our objective is to contribute to the ongoing advancement of knowledge in the management of purpura fulminans.

This infectious purpura fulminans presents as a hematological emergency, demanding early identification and prompt initiation of therapy. The comprehensive approach includes the control of precipitating factors, surgical debridement, and the provision of physiological and nutritional support, aiming to alleviate significant morbidity and mortality. The recognition of a characteristic skin rash serves as a crucial diagnostic clue, prompting immediate therapeutic intervention. Potential diagnostic delays associated with waiting for skin biopsy results may result in adverse outcomes.

Given the current lack of prospective data on the management of this condition, the efficacy of various modalities, such as hyperbaric oxygen therapy, remains uncertain. Hence, emphasizing the significance of early intervention and adopting a multidisciplinary approach is crucial for enhancing patient outcomes in cases associated with Hi-related purpura fulminans.
